# The Werner syndrome protein limits the error-prone 8-oxo-dG lesion bypass activity of human DNA polymerase kappa

**DOI:** 10.1093/nar/gku913

**Published:** 2014-10-07

**Authors:** Leena Maddukuri, Amit Ketkar, Sarah Eddy, Maroof K. Zafar, Robert L. Eoff

**Affiliations:** Department of Biochemistry and Molecular Biology, University of Arkansas for Medical Sciences, Little Rock, AR 72205-7199, USA

## Abstract

Human DNA polymerase kappa (hpol κ) is the only Y-family member to preferentially insert dAMP opposite 7,8-dihydro-8-oxo-2′-deoxyguanosine (8-oxo-dG) during translesion DNA synthesis. We have studied the mechanism of action by which hpol κ activity is modulated by the Werner syndrome protein (WRN), a RecQ helicase known to influence repair of 8-oxo-dG. Here we show that WRN stimulates the 8-oxo-dG bypass activity of hpol κ *in vitro* by enhancing the correct base insertion opposite the lesion, as well as extension from dC:8-oxo-dG base pairs. Steady-state kinetic analysis reveals that WRN improves hpol κ-catalyzed dCMP insertion opposite 8-oxo-dG ∼10-fold and extension from dC:8-oxo-dG by 2.4-fold. Stimulation is primarily due to an increase in the rate constant for polymerization (*k*_pol_), as assessed by pre-steady-state kinetics, and it requires the RecQ C-terminal (RQC) domain. In support of the functional data, recombinant WRN and hpol κ were found to physically interact through the exo and RQC domains of WRN, and co-localization of WRN and hpol κ was observed in human cells treated with hydrogen peroxide. Thus, WRN limits the error-prone bypass of 8-oxo-dG by hpol κ, which could influence the sensitivity to oxidative damage that has previously been observed for Werner's syndrome cells.

## INTRODUCTION

DNA damage is a barrier to faithful replication of the genome. Biological systems are continuously exposed to agents that react with DNA and alter its physico-chemical properties, which can impair normal nucleic acid metabolism (1,2). DNA adducts that are not repaired can block replication fork progression (3,4). The activation of replication stress response pathways can lead to the recruitment of specialized DNA polymerases (pols) to perform translesion synthesis across adducts (5). The Y-family DNA polymerases are central to this process (6). Humans have four Y-family members that participate in a number of pathways involved in cellular responses to DNA damage (6). Each human Y-family pol bears unique structural characteristic and lesion bypass properties (7). Understanding the distinct mechanism of action for each of the four human Y-family pols is an ongoing area of investigation, as the diversity of form and function found within this family of enzymes is remarkable.

The Y-family member human DNA polymerase kappa (hpol κ) was first identified as a homolog of the *dinB* gene product in *Escherichia coli* and is perhaps best recognized for its involvement in bypass of bulky minor-groove DNA adducts, as evidenced by its *in vitro* bypass properties and cellular responses to the aryl hydrocarbon receptor activation (8–10). The N-clasp of hpol κ has been proposed to help facilitate accurate and efficient bypass of *N*^2^-dG adducts (11,12). There is evidence that hpol κ contributes to bypass of other DNA adducts, as the enzyme appears to be recruited to sites of DNA damage following exposure to methylmethane sulfonate and participates in tolerance of *O*^6^-methylguanine adducts (13,14). Mis-regulation of hpol κ is observed in many tumors, including primary brain tumors (15–17). Indeed, experiments have illustrated that even moderate over-expression of hpol κ (∼2-fold) can have extremely negative consequences toward genome stability, including reduced replication fork rates, stimulation of double-strand break formation, aberrant activation of homologous recombination and increased chromosomal damage (18). Thus, studying the catalytic properties of hpol κ could provide insights into mechanisms that contribute to mutagenesis and genomic instability in human disease states, such as cancer.

An interesting feature of hpol κ catalysis is its error-prone bypass of the ubiquitous oxidative DNA lesion 7,8-dihydro-8-oxo-2′-deoxyguanosine (8-oxo-dG) (19,20). The 8-oxo-dG adduct has been widely studied because it is highly mutagenic and elevated levels of the lesion have been associated with cancer and aging (21,22). Reactive species, such as ^1^O_2_, ^·^OH, one-electron oxidants and peroxynitrate, can react with guanine to form 8-oxo-dG (23,24), and increased 8-oxo-dG levels have been linked to metastasis in several cancer types, including brain and breast cancer (25–28). It has been estimated that somewhere in the range of several hundred to a few thousand 8-oxo-dG lesions are formed in a cell per day (25,29). The most common mutations associated with 8-oxo-dG adducts are G → T transversions (30). This is due to the fact that many DNA pols readily insert deoxyadenosine monophosphate (dAMP) opposite 8-oxo-dG (31–37). The preferred mis-insertion of dAMP opposite 8-oxo-dG by hpol κ is noteworthy because it is apparently the only Y-family pol that copies the lesion in such an error-prone fashion and with essentially no reduction in catalytic efficiency (20,38–41). Structural comparisons with other Y-family pols suggest that hpol κ apparently lacks electrostatic contacts between the little finger domain and the templating 8-oxo-dG that are necessary for stabilization of *anti*-oriented 8-oxo-dG, which promotes effective pairing of dC:8-oxo-dG in other Y-family pols, such as Dpo4 from *Sulfolobus solfataricus* (42). Importantly, a recent study showed that hpol κ contributes to the mutagenic replication of 8-oxo-dG in human cells (43). Therefore, understanding hpol κ bypass of 8-oxo-dG could provide important insights into error-prone DNA replication.

Many studies have illustrated the importance of protein–protein interactions in determining the accuracy and efficiency of translesion DNA synthesis (TLS) (19,39,44–46). Interactions with the Werner syndrome protein (WRN) can stimulate hpol κ activity against 8-oxo-dG *in vitro* (47), but mechanistic features and the impact of WRN upon TLS fidelity remain unclear. The multi-functional WRN enzyme is involved in DNA replication and repair pathways, including MUTYH-mediated repair of 8-oxo-dG (48–54). Therefore, we set out to study the functional interaction between the genome caretaker WRN and Y-family member hpol κ in order to better understand the mechanism of action and potential ramifications upon translesion synthesis across DNA adducts, such as 8-oxo-dG.

## MATERIALS AND METHODS

All unlabeled deoxynucleoside triphosphates (dNTPs) were obtained from GE Healthcare Life Sciences (Piscataway, NJ, USA). All oligonucleotides used in this work were synthesized by Integrated DNA Technologies (Coralville, IA, USA) and purified using high-performance liquid chromatography (HPLC) by the manufacturer, with analysis by matrix-assisted laser desorption time-of-flight mass spectrometry. The primer sequence used for activity assays and kinetic analysis of insertion opposite dG and 8-oxo-dG was 5′-6-carboxyfluorescein (FAM)-TTTGGGGGAAGGATTC-3′ and the primer used for kinetic analysis of next-base extension was either 5′-FAM-TTTGGGGGAAGGATTCC-3′ or 5′-FAM-TTTGGGGGAAGGATTCA-3′. The template sequence used in all pol assays was 5′-TCACXGAATCCTTCCCCC-3′, where X represents either dG or 8-oxo-dG.

### Expression and purification of recombinant proteins

The hpol κ^19-526^ was expressed and purified as described previously (20). WRN constructs were expressed and purified as described previously (55), except for WRN^500-1150^, WRN^500-1092^, WRN^500-949^ and WRN^949–1092^. These WRN constructs possessed N-terminal 6xHis-glutathione transferase (GST) affinity tags. The His-GST-WRN fusion proteins were expressed in *E. coli* BL21 (DE3) Gold cells (Agilent Technologies, Santa Clara, CA, USA). Cells were grown at 37°C and 250 rpm for 3 h (OD_600_ = 0.5–0.6), followed by induction for 3 h (37°C and 250 rpm) by addition of isopropyl β-D-1-thiogalactopyranoside (1 mM), and finally harvested by centrifugation. Buffer containing 50-mM Tris-HCl (pH 7.4), 0.5-M NaCl, 10% glycerol (v/v), 5-mM β-mercaptoethanol (β-ME), lysozyme (1 mg/ml) and a protease inhibitor cocktail (Roche, Basel, Switzerland) was added to the harvested pellet. The suspension was sonicated and supernatant recovered from an ultracentrifugation step (35 000 g, 1 h, 4°C). The protein was purified by two affinity steps using Ni Sepharose (GE Healthcare Life Sciences) followed by Gluthatione Seph3rose 4B beads (GE Healthcare Life Sciences). Briefly, the protein was bound to a Ni-chelating column in 50-mM Tris-HCl (pH 7.4) buffer containing 0.3-M NaCl, 10% glycerol (v/v) and 5-mM β-ME. The column was washed with 40–60-mM imidazole and the protein eluted as a single peak in 400-mM imidazole. Following dialysis to remove imidazole and lower the concentration of NaCl to 0.15 M, the protein was added to the GST column in 25-mM Tris-HCl (pH 7.4) buffer containing 0.2-M NaCl, 10% glycerol (v/v) and 5-mM β-ME. After washing, the protein was then cleaved from the His-GST tag by treatment with HRV 3C protease (Thermo Scientific) on the GST column, according to the methods suggested by the manufacturer. The highly pure proteins (Supplementary Figure S1) were stored at –80°C in the HEPES buffer (pH 7.5) containing 0.2-M NaCl, 5-mM β-ME and 30% glycerol. The DNA binding capacity of the WRN^949-1092^ construct was analyzed as a means of verifying proper folding of this short construct (Supplementary Figure S2).

### Full-length extension polymerase assays

Fluorescein-labeled primer was annealed to template oligonucleotide by heating a 1:2 molar ratio of primer:template DNA (p/t-DNA) to 95°C for 5 min and then slow cooling to room temperature (RT). The p/t-DNA was then incubated with hpol κ prior to extension in the presence or absence of WRN. For full-length extension experiments, hpol κ was added to the tube first, followed by addition of WRN (where indicated). The DNA substrate was added after WRN and the solution was incubated on ice for 30 min prior to starting the reaction. Each reaction was initiated by adding dNTP·MgCl_2_ (0.25 mM of each dNTP and 5-mM MgCl_2_) solution to a pre-incubated hpol κ·DNA complex (2-nM hpol κ and 200-nM DNA). Where indicated WRN (100 nM) was incubated with hpol κ and DNA. Unless otherwise stated, all enzymatic reactions were carried out at 37°C in 50-mM HEPES buffer (pH 7.5) containing 60-mM KCl, 5-mM dithiothreitol, 100-μg ml^−1^ bovine serum albumin (BSA) and 10% (v/v) glycerol. At the indicated time points, 4-μl aliquots were quenched with 16 μl of a 95% formamide (v/v)/20-mM ethylenediaminetetraacetic acid (EDTA)/0.1% bromophenol blue (w/v) solution and were separated by electrophoresis on a 16% polyacryamide (w/v)/7-M urea gel. The products were then visualized using a Typhoon imager (GE Healthcare Life Sciences) and quantified using ImageQuant^TM^ software (GE Healthcare Life Sciences). The total product formed over time was fit to a single-exponential equation [Equation (1)]:

(1) \begin{equation*} y = A(1 - e^{ - k_{{\rm obs}} t} ), \end{equation*}

where *A* is the product formed, *k*_1_ is the rate constant defining polymerization under the conditions used for the experiment being analyzed and *t* is the time.

### Steady-state kinetic analysis of polymerase activity

Single-nucleotide incorporation by hpol κ (2 nM) on DNA substrates (200 nM) was measured over a range of dNTP concentrations (0–250 μM). The reaction buffer and the general protocol was the same as that used for full-length extension assays. Time points were chosen such that only dNTP insertion by hpol κ^19-526^ (and not exonucleolytic degradation of the primer) was observed for reactions with either full-length WRN (a.a. 1-1432) or the WRN exo domain (a.a. 1-333). Products were analyzed as described for full-length extension assays. The initial portion of the velocity curve was fit to a linear equation in the program GraphPad Prism (GraphPad, San Diego, CA, USA). The resulting velocity was plotted as a function of dNTP concentration and then fit to a hyperbolic equation, correcting for enzyme concentration, to obtain estimates for the turnover number (*k*_cat_) and Michaelis constant (*K*_M,dNTP_).

### Pre-steady-state kinetic analysis of hpol κ polymerase activity

All pre-steady-state experiments were performed using a KinTek RQF-3 model chemical quench-flow apparatus (KinTek Corp., Austin, TX, USA). The hpol κ^19–526^ (25 nM) enzyme was pre-incubated with FAM-labeled p/t-DNA (50 nM). The reaction was initiated by rapid mixing of the enzyme·DNA solution with a solution containing MgCl_2_ (5 mM) and varying concentrations of dNTP (from 0.5 μM to 1 mM). Polymerase catalysis was stopped by the addition of 50-mM EDTA (pH 9.0). Product formation was either fit to a single-exponential equation [Equation (1)] or to a single-exponential equation with a second linear phase [Equation (2)]:

(2) \begin{equation*} y = A(1 - e^{ - k_{{\rm obs}} t} ) + k_2 t, \end{equation*}

where *A* is the product formed in the burst phase, *k*_1_ is the rate constant defining polymerization under the conditions used for the experiment being analyzed, *k*_2_ is the observed second-order rate of product formation and *t* is the time. Equation (1) was only used to fit pre-steady-state results for low concentrations of dNTP (0.5–1-μM dNTP) where the second phase of the reaction curve was not apparent.

### Measurement of WRN 3′-5′ exonuclease activity

Exonucleolytic degradation of polymerase products was measured by incubating WRN exo (a.a. 1-333; 100 nM) with the indicated 14/18-mer DNA substrates (200 nM). For experiments measuring WRN exo activity, p/t-DNA (200 nM) was added to the reaction tube followed by addition of WRN^1-333^. The solution was allowed to incubate for ∼30 min and then initiated by addition of MgCl_2_ (5 mM). For experiments to test competing exo and pol activities, hpol κ (2 nM) was added to the reaction mixture first, followed by DNA (200 nM) and finally, WRN^1-333^. The mixture was allowed to incubate for ∼30 min before initiating the reaction by addition of dGTP (100 μM) and MgCl_2_ (5 mM). In both sets of experiments, a zero time point was collected by adding quench to the reaction mixture prior to the addition of dGTP/Mg^2+^. Reactions were quenched, products separated and quantified in a manner identical to that described above for the polymerase assays. WRN-catalyzed degradation of FAM-labeled 14-mer primer was quantified and the rate of degradation was estimated by linear regression analysis. The sum of all degraded products was measured to estimate the rate of WRN action. To quantify exo activity in reactions containing hpol κ, we quantified the substrate, pol products and exo products as total DNA and then calculated the amount of substrate degraded by WRN as the fraction of total DNA in the lane. The rate of exo product formation was estimated by fitting the initial portion of the velocity curve to a linear function.

### Pull-down assays with recombinant WRN and hpol κ

Glutathione Sepharose 4 Fast Flow beads (GE Healthcare) were washed extensively in binding 25-mM HEPES (pH 7.5) buffer containing 0.1-M NaCl, 10% (v/v) glycerol and 5-mM β-ME. Two sets of beads were prepared for the pull-down experiments. The washed beads were incubated with either purified GST protein or purified GST-tagged hpol κ^19-526^. Both sets of beads were incubated at 4°C overnight with constant agitation in the binding buffer. The beads were again washed thoroughly with the binding buffer to remove unbound protein and used for subsequent pull-down experiments with the different WRN constructs. GST-coated beads served as the negative control for all experiments testing specific pull-down of WRN constructs by hpol κ^19-526^. For each experiment, 10 μl of protein-coated beads (∼1200-pmol protein) were mixed with the WRN construct (WRN^1-333^, WRN^949-1092^, WRN^500-949^ and WRN^500-1092^) in a total volume of 500-μl binding buffer for a final WRN protein concentration of 1 μM (∼500-pmol protein). The tubes were incubated for 16 h at 4**°**C with constant agitation, after which they were centrifuged at 5000 rpm for 5 min. The supernatant, which represented unbound fraction, was collected, centrifuged under vacuum to dryness and re-suspended in 40 μl of dH_2_O. The beads were then washed three times with 1 ml of binding buffer each time. Washed beads were then boiled in 20 μl of sodium dodecyl sulphate-polyacrylamide gel electrophoresis (SDS-PAGE) loading buffer for 10 min to elute bound proteins and then diluted to 40 μl with 1X phosphate buffered saline (PBS). Both the unbound fractions (loaded 20 μl, 50% of total supernatant) and bound proteins eluted from the beads (loaded 20 μl, 50% of total eluted protein) were separated on a 4–20% gradient SDS-PAGE and stained with Coomassie Brilliant Blue for visualization. The stained gels were scanned on an ImageQuant LAS 4000 scanner (GE Healthcare), and the band intensities were quantified using ImageQuant software. For each experiment, the fraction of WRN protein bound to the beads was calculated by dividing the band intensity of protein eluted from the beads by total intensity (obtained by adding intensities in unbound supernatant and the eluted lanes). Specific binding of each WRN protein to hpol κ^19-526^ was expressed as a ratio of fraction bound to the GST-tagged hpol κ^19-526^ beads divided by the fraction bound to the GST-coated beads.

### Immunofluorescence assays for determining the co-localization of WRN and hpol κ

HeLa cells used for this study were kindly provided by Dr Timothy Chambers (University of Arkansas for Medical Sciences, Little Rock, AR) and maintained in Dulbecco's minimum essential medium (Life technologies, Grand Island, NY, USA) containing 10% (v/v) heat-inactivated fetal bovine serum (Life technologies) and supplemented with penicillin and streptomycin (Life technologies). For Immunofluorescence studies, HeLa cells (0.3 × 10^6^) cultured on glass coverslips were treated with 0.5 mM H_2_O_2_ (Sigma) for 4 h. Untreated cells were prepared as a control. After 4 h, cells were fixed with 3.7% (v/v) formaldehyde for 20 min at RT. The fixed cells were then permeabilized and blocked by soaking in 0.2% (v/v) Triton X-100 and PBS containing 5 mg/ml BSA for 30 min at RT. The fixed and permeabilized cells were then incubated for 1 h at RT with primary antibodies diluted in blocking solution: rabbit anti-WRN and mouse anti-hpol κ (Novus biological, 1:100). The slides were washed with PBS and incubated for 1 h at RT with secondary antibodies diluted in blocking solution: Dylight 488 conjugated goat anti-mouse IgG (Thermo Scientific Pierce, Rockford, IL, USA; 1:100) and Dylight 550 conjugated goat anti-rabbit IgG (Thermo Scientific Pierce, Rockford, IL, USA; 1:100). After washing with PBS, coverslips were mounted on Vectashield (Vector Laboratories) containing 4′,6-diamidino-2-phenylindole (DAPI) and images were captured on an Olympus IX81 fluorescence microscope. Cells possessing one or more yellow foci were scored as positive for WRN/hpol κ co-localization. At least 50 nuclei were scored in each of three independent experiments.

## RESULTS

### The exonuclease and RecQ C-terminal domains of WRN are required for full stimulation of hpol κ primer extension properties

**Figure 1.**
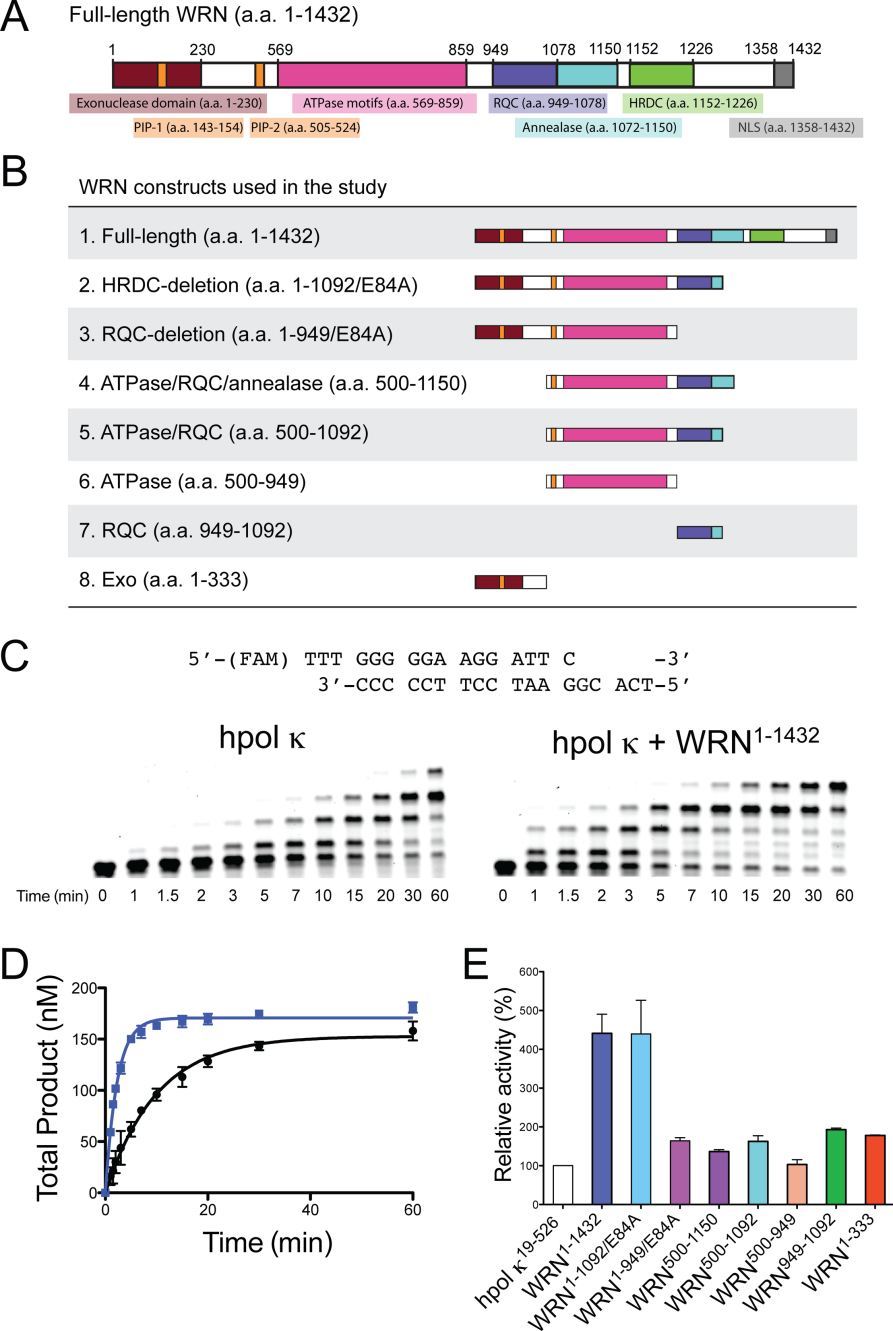
WRN stimulates hpol κ^19-526^ activity on undamaged DNA templates. (**A**) An overview of the full-length WRN protein showing domains with either structural or catalytic properties relevant to the current study. HRDC: Helicase and RNaseD C-terminal; NLS: nuclear localization signal; PIP: PCNA interacting peptide; RQC: RecQ C-terminal; the E84A mutation abrogates WRN exonuclease activity. (**B**) Schematic illustration of the eight WRN constructs utilized in the study. (**C**) DNA synthesis by hpol κ^19-526^ (2 nM) was monitored over time using a 13/18-mer primer-template DNA substrate (200 nM) in the absence of WRN and in the presence of full-length WRN^1-1432^ (100 nM). A schematic of the p/t-DNA substrate is shown above the gel results. (**D**) Total product formation [i.e. all of the product bands from panel (C)] was plotted as a function of time. The mean ± SEM is shown (*n* = 2). Product formation in each experiment was fit to a single-exponential equation [Equation (1)] to yield the following kinetic parameters: No WRN (black closed circles, •): *A* = 153 ± 4 nM, *k*_obs_ = 0.099 ± 0.007 min^−1^; WRN^1-1432^ (blue closed squares, 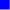): *A* = 171 ± 2 nM, *k*_obs_ = 0.43 ± 0.01 min^−1^. (**E**) The relative activity of hpol κ in the presence of different WRN constructs is shown. Polymerase extension assays with additional WRN constructs were performed as described in panel (C). The rate constants for the total product formed were 0.43 ± 0.02 (hpol κ + WRN^1-1092/E84A^), 0.16 ± 0.01 (hpol κ + WRN^1-949/E84A^), 0.13 ± 0.01 (hpol κ + WRN^500-1150^), 0.18 ± 0.01 (hpol κ + WRN^500-1092^), 0.094 ± 0.008 (hpol κ + WRN^500-949^), 0.18 ± 0.01 (hpol κ + WRN^949-1092^) and 0.19 ± 0.01 (hpol κ + WRN^1-333^) nM min^−1^. The values reported represent the mean ± SEM (*n* = 2). The relative activity of hpol κ was calculated by dividing the rate constant for primer extension in the presence of the WRN construct by the rate constant for primer extension by hpol κ alone then multiplying by 100.

WRN is a protein with several enzymatic functions and multiple domains (Figure 1A) (48). We began our study by testing the ability of full-length WRN and a series of WRN truncation mutants to stimulate extension by hpol κ on unmodified DNA templates (Figure 1B). All WRN stocks were checked for contaminating polymerase activity by performing extension experiments out to an hour in the absence of hpol κ (data not shown). Extension assays were repeated at least twice to ensure the reproducibility of any stimulation of polymerization by WRN. Total product formed in each lane was plotted as a function of time and the resulting curve was fit to a single-exponential equation to obtain an estimate of the rate constant for primer extension by hpol κ. To compare the relative effect of different WRN constructs on hpol κ extension activity, we divided the rate constant for primer extension in the presence of WRN by the rate constant for primer extension when hpol κ is alone and multiplying by a factor of one hundred to yield percent activity. In this way, we were able to make quantitative comparisons for stimulation of pol extension activity by different WRN constructs.

Similar to our previous results and those reported by the Loeb group with hpol η (47,55), we find that full-length WRN stimulates hpol κ extension activity on unmodified template DNA (Figure 1C). The rate constant for total product formation is increased ∼4-fold by inclusion of full-length WRN^1-1432^ (Figure 1D). We then went on to test the domain requirements for WRN-mediated stimulation of hpol κ activity by performing extension assays with truncated versions of the WRN protein. We find that the Helicase and RNaseD C-terminal (HRDC) deletion construct (a.a. 1-1092/E84A) stimulates hpol κ extension activity ∼4-fold, similar to full-length WRN (Figure 1E and Supplementary Figure S3). As we observed previously with hpol η (55), deletion of the RecQ C-terminal (RQC) domain (a.a. 1-949/E84A) reduces the effect upon hpol κ to a little less than 2-fold enhancement. A similar reduction in the level of pol stimulation was observed for a WRN construct possessing the ATPase/RQC/annealase domains (a.a. 500-1150), indicating a need for both RQC and exo domains for full stimulation of hpol κ extension activity. The WRN ATPase/RQC construct (a.a. 500-1092) stimulated hpol κ activity 2-fold but the WRN ATPase domain alone (a.a. 500-949) did not stimulate extension at all (Figure 1E). Adding either the WRN exo domain (a.a. 1-333) or the RQC domain (a.a. 949-1092) to the reaction mixture results in an ∼2-fold stimulation of hpol κ extension activity (Figure 1E). These results indicate that both the WRN exo and RQC domains are important for stimulation of hpol κ extension activity, consistent with results obtained with hpol η (55).

### Physical interaction between WRN and hpol κ

The pol extension results showed that WRN stimulates the activity of the hpol κ catalytic core and that full stimulation required both exo and RQC domains of WRN. These results imply that the two proteins bind to one another but there is no previous evidence for a physical interaction between WRN and hpol κ. To determine if WRN and hpol κ physically interact with one another, we performed *in vitro* pull-down experiments with recombinant enzymes. A GST-tagged version of hpol κ (GST-hpol κ) was incubated with glutathione sepharose beads and WRN. The specificity of the interaction with hpol κ was confirmed by incubating each WRN construct with GST-coated beads (i.e. no hpol κ present in the binding mixture). We used four WRN constructs to investigate the interaction with hpol κ: (i) WRN^500-1092^ ATPase and RQC domains, (ii) WRN^500-949^ ATPase domain, (iii) WRN^949-1092^ RQC domain and (iv) WRN^1-333^ exo domain.

**Figure 2.**
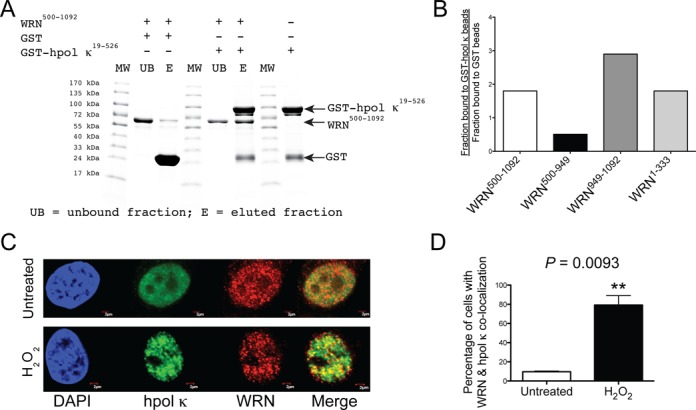
WRN and hpol κ physically interact and co-localize in cells following exposure to hydrogen peroxide. (**A**) Recombinant hpol κ possessing a glutathione transferase (GST) affinity tag (GST-hpol κ; 1200 pmol) was bound to glutathione (GSH)-coated beads and then incubated with WRN^500-1092^ (500 pmol). In a separate experiment, WRN^500-1092^ was incubated with GST-bound beads (i.e. no hpol κ present). The unbound fraction was removed from the beads; the beads were washed and bound proteins eluted by boiling in SDS-PAGE buffer. The unbound fraction and the eluted proteins were separated by SDS-PAGE and visualized with Coomassie blue. (**B**) The amount of WRN that bound GST-hpol κ-coated beads in a specific manner was quantified (see the Materials and Methods section for a description of the calculation). Specific binding to GST-hpol κ was observed for WRN RQC-containing constructs (a.a. 500-1092 and a.a. 949-1092) and for the WRN exo domain (a.a. 1-333). Specific binding was not observed for the WRN ATPase domain (a.a. 500-949). (**C**) WRN and hpol κ co-localize at nuclear foci following exposure to oxidative stress. HeLa cells grown on glass coverslips were either left untreated or were treated with H_2_O_2_ (0.5 mM, 4 h). The cells were fixed and immunostaining was performed with antibodies against hpol κ (green) and WRN (red). DAPI staining of the nucleus is also shown (blue). Co-localization of hpol κ and WRN is shown as yellow in the merged images. The scale bar represents 2 μm. (**D**) Quantification of WRN and hpol κ nuclear foci formation in untreated and H_2_O_2_-treated cells. A minimum of 50 cells were scored from three independent experiments. The mean (± SD) is shown. **P < 0.01.

The WRN^500-1092^ ATPase/RQC construct bound to GST-hpol κ-coated beads nearly 2-fold better than the negative control GST beads (Figure 2A). Likewise, the WRN exo domain (a.a. 1-333) was found to interact specifically with GST-hpol κ-coated beads (Figure 2B). The strongest interaction was observed with the WRN RQC domain (a.a. 949-1092), which was enriched almost 3-fold over the negative control GST-coated beads (Figure 2B). Of the four constructs tested, only the WRN^500-949^, which lacks both the RQC and exo domains, failed to bind specifically to GST-hpol κ-coated beads (Figure 2B). These results confirm that WRN and hpol κ physically interact through both the RQC and exo domains, consistent with the pol extension results and similar to our previous study with WRN and hpol η (55).

We then went on to investigate whether WRN and hpol κ co-localize inside cells in response to DNA damage. To examine the potential cellular interaction between the two enzymes, we treated HeLa cells with H_2_O_2_ (500 μM) for up to 4 h and used immunofluorescence to monitor the spatial distribution of WRN and hpol κ. In untreated cells, there is very little overlap in the signal for hpol κ and WRN (Figure 2C). Treatment with H_2_O_2_ results in an increased number of discrete foci for both enzymes and an increase in co-localization (Figure 2C). The number of cells with WRN and hpol κ co-localized increases from less than 10% in untreated cells to almost 80% of cells showing overlap following exposure to H_2_O_2_ (Figure 2C and D). The finding that WRN and hpol κ are found co-localized in response to oxidative damage is the first to show that these two enzymes might encounter each other inside human cells. We then went on to examine the mechanism of WRN action on TLS by hpol κ.

### Steady-state kinetic analysis of WRN-mediated stimulation of hpol κ activity on undamaged DNA

To ascertain whether WRN altered single-nucleotide insertion kinetics for hpol κ, we measured the turnover number (*k*_cat_) and the Michealis constant (*K*_M,dNTP_) for hpol κ-catalyzed single-nucleotide insertion reactions. From these values we calculated the specificity constant (*k*_cat_/*K*_M,dNTP_), which is often considered an estimate of catalytic efficiency and we use the two terms (i.e. specificity constant and catalytic efficiency) interchangeably throughout the text. We first examined steady-state kinetics for dCTP insertion opposite unmodified template dG. Similar to extension assays, those WRN constructs possessing the RQC domain appear to impart the largest stimulatory effect on the *k*_cat_ for hpol κ-catalyzed insertion of deoxycytidine monophosphate (dCMP) opposite template dG (Table 1). The increase in the specificity constant is highest (∼3.1-fold) for the full-length WRN protein. Notably, WRN^1-1432^ does not alter the kinetic parameters for mis-insertion of dAMP opposite template dG (Table 1). The HRDC-deletion construct (a.a. 1-1092/E84A) increases the specificity constant for hpol κ-catalyzed insertion of dCMP opposite dG ∼2.8-fold, whereas the other two RQC-containing WRN constructs (a.a. 500-1150 and a.a. 949-1092) only increase the specificity constant ∼2-fold, consistent with the extension results. There is a very slight increase in the *k*_cat_ for hpol κ insertion of dCMP opposite dG in the presence of WRN^1-333^ and WRN^1-949/E84A^, but there is essentially no change in the specificity constant for either of the constructs lacking the RQC (Table 1). As such, the WRN RQC domain appears to be a stronger factor in stimulating the efficiency of single-nucleotide insertion by hpol κ on undamaged templates.

**Table 1.** Steady-state kinetic parameters of accurate base insertion by hpol κ opposite dG in the presence of WRN

	*k*_cat_ (min^−1^)	*K*_M,dNTP_ (μM)	*k*_cat_/*K*_M,dNTP_ (min^−1^ μM^−1^)
dCMP insertion opposite dG			
No WRN	14.6 ± 0.4	1.3 ± 0.2	11.2
WRN^1-1432^	41.7 ± 1.3	1.2 ± 0.2	34.7
WRN^1-1092/E84A^	46.2 ± 2.1	1.5 ± 0.4	30.8
WRN^1-949/E84A^	18.6 ± 0.5	1.6 ± 0.3	11.6
WRN^500-1150^	27.0 ± 1.0	1.2 ± 0.2	22.5
WRN^949-1092^	23.2 ± 1.1	1.1 ± 0.4	21.1
WRN^1-333^	16.3 ± 0.5	1.2 ± 0.3	13.6
dAMP insertion opposite dG			
No WRN	0.32 ± 0.02	24.5 ± 7.8	0.013
WRN^1-1432^	0.35 ± 0.04	23.7 ± 7.5	0.015

For the determination of kinetic values, graphs of product formation versus dNTP concentration were plotted using nonlinear regression analysis (one-site hyperbolic fit) in the GraphPad prism program. The standard error of the fit is reported for each value.

### Steady-state kinetic analysis of WRN-mediated stimulation of hpol κ activity on 8-oxo-dG-containing template DNA

We were interested in examining whether WRN could modulate the DNA damage bypass properties of hpol κ. A previous study showed qualitative stimulation of hpol κ-catalysis against several DNA adducts (47), but the effect upon the efficiency and accuracy of polymerization remains unclear. While the TLS properties of hpol κ have been studied with a variety of adducts, we chose to investigate 8-oxo-dG for three reasons: (i) hpol κ is by far the most error-prone Y-family member at bypass of 8-oxo-dG, (ii) there is evidence that hpol κ contributes to mutagenic bypass of 8-oxo-dG in human cells and (iii) crystal structures are available with hpol κ in ternary complex with 8-oxo-dG-modified DNA. In this way, the results obtained with hpol κ in the presence of WRN can be assessed within the context of rigorous mechanistic studies and could provide insights into cellular mechanisms of mutagenesis.

We performed steady-state analysis of hpol κ-catalyzed single-nucleotide insertion across from 8-oxo-dG. Single-nucleotide insertion kinetics reveal that full-length WRN^1–1432^ stimulates the *k*_cat_ for dCMP insertion opposite 8-oxo-dG a little over 3-fold (Table 2). The addition of full-length WRN also decreases the *K*_M,dNTP_ for dCTP ∼3-fold. Importantly, the addition of WRN increases the specificity constant for dCMP insertion opposite 8-oxo-dG ∼10-fold relative to that observed for hpol κ alone. The WRN HRDC-deletion construct (a.a. 1-1092/E84A) also stimulates the specificity constant for dCMP insertion opposite 8-oxo-dG over 10-fold. The ATPase/RQC (a.a. 500-1150) and RQC (a.a. 949-1092) constructs increase the efficiency of dCMP insertion opposite 8-oxo-dG over 4- and 5-fold, respectively. However, kinetic analysis of hpol κ-catalyzed insertion of dCMP opposite 8-oxo-dG in the presence of WRN exo (a.a. 1-333) revealed no change in the specificity constant. Thus, it would appear that the RQC domain is primarily responsible for WRN-mediated enhancement of hpol κ insertion of dCMP opposite 8-oxo-dG.

**Table 2.** Steady-state kinetic parameters of accurate base insertion by hpol κ opposite 8-oxo-dG in the presence of WRN

dCTP opposite 8-oxo-dG	*k*_cat_ (min^−1^)	*K*_M,dNTP_ (μM)	*k*_cat_/*K*_M,dNTP_ (min^−1^ μM^−1^)
No WRN	12.4 ± 0.7	23.4 ± 3.1	0.5
WRN^1-1432^	38.5 ± 2.2	7.4 ± 1.7	5.2
WRN^1-1092/E84A^	36.3 ± 0.8	6.3 ± 0.6	5.8
WRN^1-949/E84A^	12.4 ± 1.6	42.9 ± 13.0	0.3
WRN^500-1150^	35.3 ± 3.5	16.2 ± 5.4	2.2
WRN^949-1092^	34.5 ± 4.2	12.7 ± 5.6	2.7
WRN^1-333^	10.8 ± 0.2	21.5 ± 1.6	0.5

Kinetic parameters were obtained as indicated in Table 1.

We then examined hpol κ-catalyzed mis-insertion of dAMP opposite 8-oxo-dG. Normally, wild-type hpol κ catalyzes the mis-insertion of dAMP opposite 8-oxo-dG ∼10- to 15-fold more effectively than it does dCMP insertion opposite 8-oxo-dG (20,56). Adding full-length WRN^1-1432^ to the reaction mixture only increases the specificity constant for dAMP insertion opposite 8-oxo-dG 1.3-fold (Table 3), much less than the effect observed for dCMP insertion. A similar lack of an effect is observed for the other WRN constructs, the exception being the WRN exo domain. The addition of the WRN^1-333^ exo domain results in a diminished *k*_cat_ for dAMP insertion opposite 8-oxo-dG, but the specificity constant increases slightly because of a reduced Michaelis constant. Based on the larger changes observed for dCMP insertion opposite 8-oxo-dG, it does not seem as though WRN has an impact on hpol κ-catalyzed mis-insertion of dAMP opposite 8-oxo-dG. The major conclusion from these results is that the addition of WRN improves the fidelity of hpol κ catalysis opposite 8-oxo-dG since it shifts the balance from an ∼14-fold preference for dAMP insertion opposite 8-oxo-dG to a less than 2-fold preference for error-prone nucleotide selection.

**Table 3.** Steady-state kinetic parameters of mis-insertion by hpol κ opposite 8-oxo-dG in the presence of WRN

dATP opposite 8-oxo-dG	*k*_cat_ (min^−1^)	*K*_M,dNTP_ (μM)	*k*_cat_/*K*_M,dNTP_ (min^−1^ μM^−1^)
No WRN	19.3 ± 0.6	2.8 ± 0.5	6.9
WRN^1-1432^	17.9 ± 0.8	2.0 ± 0.5	8.9
WRN^1-1092/E84A^	18.2 ± 0.6	1.9 ± 0.4	9.5
WRN^500-1150^	22.1 ± 2.0	3.9 ± 1.1	5.6
WRN^949-1092^	20.0 ± 1.3	4.0 ± 1.1	5.0
WRN^1-333^	13.2 ± 0.5	1.5 ± 0.4	8.8

Kinetic parameters were obtained as indicated in Table 1.

### Pre-steady-state kinetic analysis of WRN-mediated stimulation of hpol κ activity on 8-oxo-dG-containing template DNA

The improved fidelity of hpol κ opposite 8-oxo-dG observed in the steady-state kinetic assays was primarily driven by WRN-mediated stimulation of the *k*_cat_ value for dCMP insertion opposite 8-oxo-dG. We next sought to understand whether the altered *k*_cat_ was due to an increased rate constant for polymerization (*k*_pol_), as we observed previously for hpol η on unmodified DNA (55). To investigate this possibility, we performed pre-steady-state kinetic analysis of hpol κ-catalyzed insertion of either dCMP or dAMP opposite 8-oxo-dG in either the presence or absence of full-length WRN.

**Figure 3.**
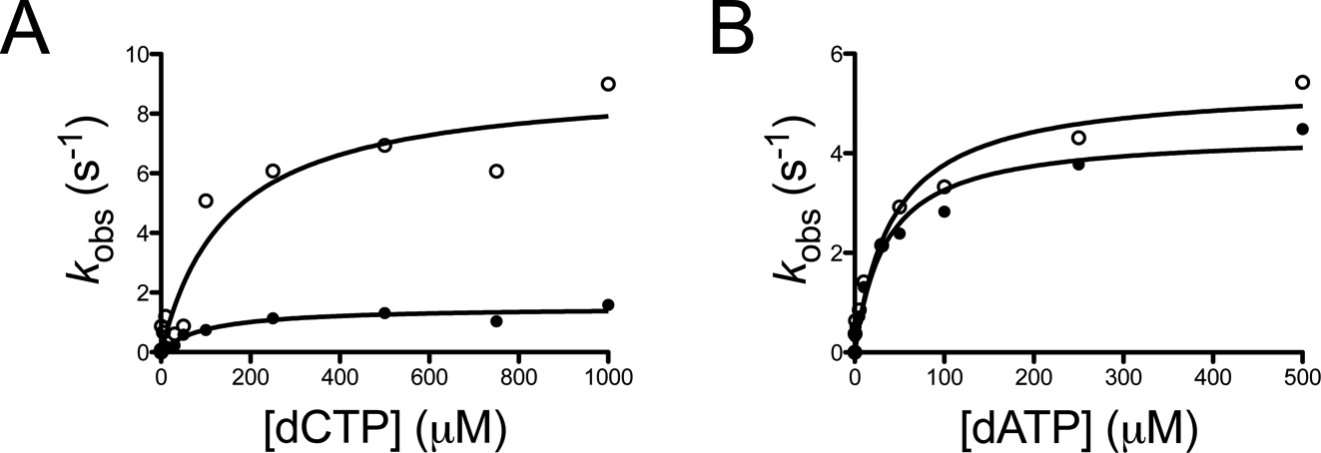
WRN stimulates the pre-steady-state rate constant *k*_pol_ for dCMP insertion opposite 8-oxo-dG by hpol κ. (**A**) hPol κ-catalyzed (25 nM) incorporation of dCTP opposite 8-oxo-dG-containing p/t-DNA (50 nM) was performed in the presence of WRN^1-1432^ (250 nM) and varying concentrations of dCTP (0.5-1000 μM). The product curves were fit to Equation (2) except for the experiment with 0.5 μM, which was fit to Equation (1). The observed rate of product formation (*k*_obs_) was plotted as a function of [dCTP] for experiments with hpol κ alone (•) and for experiments with hpol κ performed in the presence of WRN^1-1432^. The resulting kinetic parameters are listed in Table 4. (**B**) hPol κ-catalyzed (25 nM) incorporation of dATP opposite 8-oxo-dG-containing p/t-DNA (50 nM) was performed in the presence of WRN^1-1432^ (250 nM) and varying concentrations of dATP (0.5-500 μM). The product curves were fit to Equation (2). The observed rate of product formation (*k*_obs_) was plotted as a function of [dATP] for experiments with hpol κ alone (•) and for experiments with hpol κ performed in the presence of WRN^1-1432^. The resulting kinetic parameters are listed in Table 4.

Pre-steady-state experiments were performed under enzyme-limiting concentrations with varying concentrations of either dCTP or dATP (Figure 3). The observed rate constant (*k*_obs_) was plotted as a function of dNTP concentration in order to determine *k*_pol_ and *K*_d,dNTP_ for both error-prone and error-free insertion across from 8-oxo-dG. The addition of WRN stimulates *k*_pol_ for hpol κ-catalyzed insertion of dCMP opposite 8-oxo-dG 6-fold but does not greatly alter the affinity of the incoming nucleotide (Figure 3A and Table 4). The efficiency of the reaction (*k*_pol_/*K*_d,dNTP_) is increased 4-fold for dCMP insertion opposite 8-oxo-dG. The addition of WRN does not alter the efficiency of dAMP insertion by hpol κ (Figure 3B). The overall efficiency of the mis-insertion reaction is essentially the same whether WRN is present or not (Table 4). From these results, we conclude that WRN stimulates the rate constant *k*_pol_ value for hpol κ-catalyzed insertion of dCMP opposite 8-oxo-dG.

**Table 4.** Pre-steady-state kinetic parameters for nucleotide insertion opposite 8-oxo-dG by hpol κ in the presence and absence of WRN

	DNA substrate	*k*_pol_ (s^−1^)	*K*_d,dNTP_ (μM)	*k*_pol_/*K*_d,dNTP_ (s^−1^ μM^−1^)
No WRN	dCTP:8-oxo-dG	1.5 ± 0.1	100 ± 30	0.015
WRN^1-1432^	dCTP:8-oxo-dG	9.1 ± 1.1	149 ± 61	0.061
No WRN	dATP:8-oxo-dG	4.4 ± 0.3	35 ± 8	0.12
WRN^1-1432^	dATP:8-oxo-dG	5.4 ± 0.4	42 ± 10	0.13

The observed rate constant for dNTP insertion (*k*_obs_) was plotted as a function of dNTP concentration and fit to a quadratic equation. The standard error of the fit is reported for each value.

### The effect of WRN on hpol κ-catalyzed next-base extension past 8-oxo-dG

Previous studies indicate that hpol κ extends dA:8-oxo-dG base pairs (bp) about 2-fold more efficiently than it does dC:8-oxo-dG (56). The preference for extending from a mis-paired dA:8-oxo-dG was largely due to an increase in the Michaelis constant for extension from dC:8-oxo-dG bp relative to that for dA:8-oxo-dG. We wanted to determine if WRN could modulate extension by hpol κ in a way that might alter the fidelity of TLS across 8-oxo-dG.

We measured the steady-state kinetic parameters for hpol κ-catalyzed extension from four different bp: (i) dC:dG, (ii) dA:dG, (iii) dC:8-oxo-dG and (iv) dA:8-oxo-dG. The template base for these p/t-DNA substrates was dC. Accordingly, insertion of dGMP was measured with the four different primer-template substrates in the presence and absence of full-length WRN. As with the other steady-state experiments, we did not observe WRN^1-1432^ exonuclease activity under the conditions used to measure single-nucleotide insertion by hpol κ (Supplementary Figure S4). For unmodified DNA substrates, we find that WRN stimulates hpol κ-catalyzed extension from dC:dG bp but not from dA:dG mis-pairs substrates (Table 5). We observe a slightly different effect for 8-oxo-dG containing DNA substrates. Interestingly, the inclusion of full-length WRN leads to an increase in the *k*_cat_ for extension from dC:8-oxo-dG bp, with little or no effect on the *K*_M,dNTP_ (Table 5). In this way, WRN increases the efficiency for extension from the dC:8-oxo-dG bp about 2.4-fold. The *k*_cat_ for extension from dA:8-oxo-dG is decreased in the presence of WRN, while the *K*_M,dNTP_ is increased, which results in a little over 2-fold reduction in the specificity constant (*k*_cat_/*K*_M,dNTP_) for extension from the mis-pair relative to extension by hpol κ alone (Table 5). The net effect of WRN on pol fidelity is an approximate 5-fold increase in the accuracy of hpol κ extension from 8-oxo-dG containing substrates since accurate extension is improved 2.4-fold while extension of dA:8-oxo-dG is decreased 2-fold.

**Table 5.** Steady-state kinetic parameters for next-base extension by hpol κ in the presence and absence of WRN for templates containing either dG or 8-oxo-dG

	DNA substrate^a^	*k*_cat_ (min^−1^)	*K*_M,dNTP_ (μM)	*k*_cat_/*K*_M,dNTP_ (min^−1^ μM^−1^)
No WRN	dC:dG	13.7 ± 0.4	7.0 ± 0.7	1.9
WRN^1-1432^	dC:dG	26.6 ± 0.5	8.0 ± 0.6	3.3
No WRN	dA:dG	0.10 ± 0.01	3.8 ± 1.0	0.026
WRN^1-1432^	dA:dG	0.11 ± 0.01	4.6 ± 1.5	0.024
No WRN	dC:8-oxo-dG	8.0 ± 2.0	140 ± 60	0.057
WRN^1-1432^	dC:8-oxo-dG	18.0 ± 1.0	130 ± 10	0.14
No WRN	dA:8-oxo-dG	16.5 ± 1.0	56 ± 8	0.29
WRN^1-1432^	dA:8-oxo-dG	14.0 ± 0.7	105 ± 18	0.13

^a^All next-base extension experiments measured dGTP insertion since the next template base was dC. The standard error of the fit is reported for each value.

### Proofreading activity of the WRN exonuclease domain

**Figure 4.**
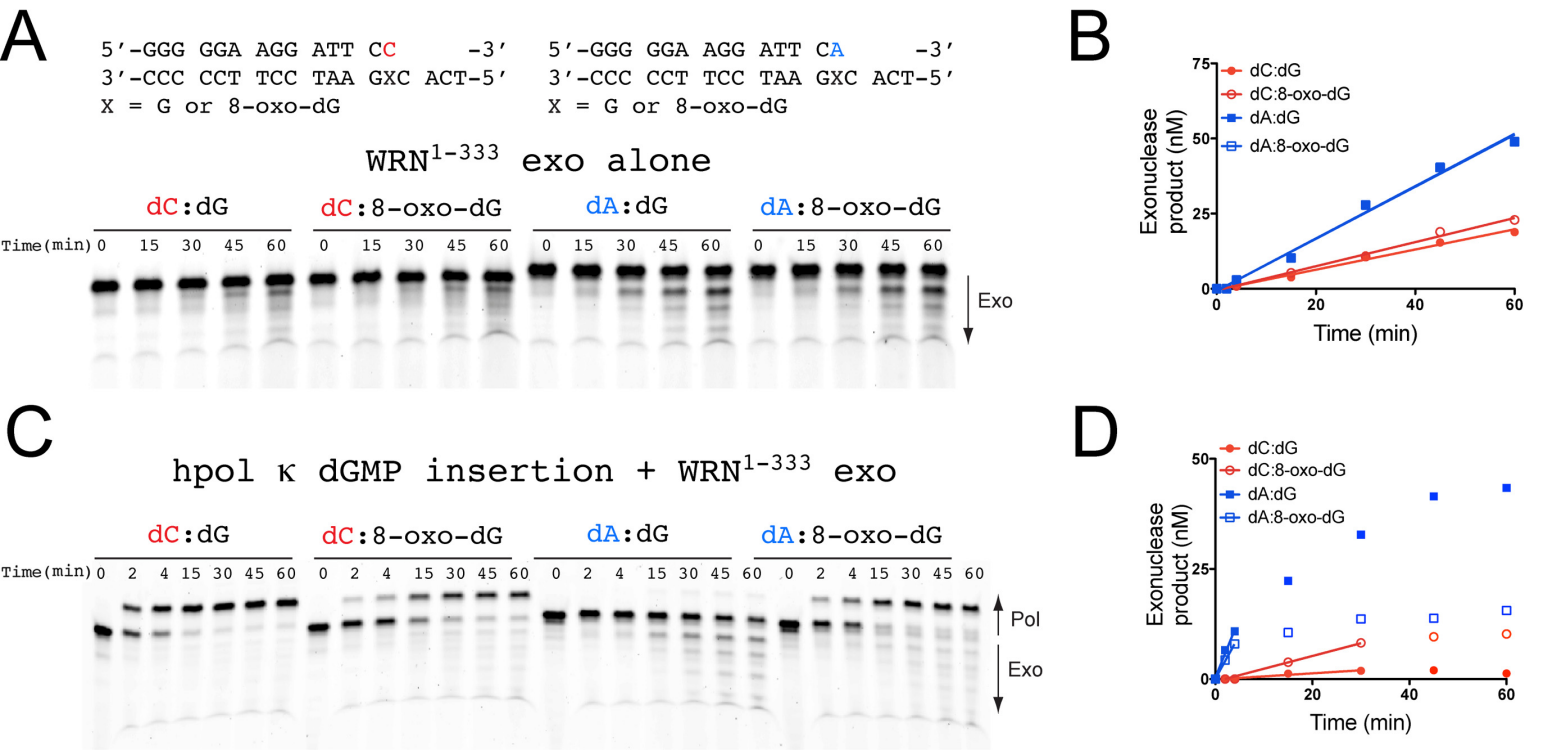
The WRN exonuclease domain preferentially degrades dA:8-oxo-dG mis-pairs. (**A**) WRN exo (100 nM) activity was measured for 14/18-mer DNA substrates (200 nM) possessing the terminal bp indicated above the gel image. (**B**) WRN-catalyzed exonucleolytic degradation of primer was quantified and plotted as a function of time. The sum of all degraded product bands was quantified. The data were analyzed by linear regression to estimate the rate of primer degradation for the linear portion of the velocity curve: dC:dG (closed circles): *v*_obs_ = 0.33 ± 0.02 nM min^−1^; dC:8-oxo-dG (open circles): *v*_obs_ = 0.40 ± 0.01 nM min^−1^; dA:dG (closed squares): *v*_obs_ = 0.87 ± 0.04 nM min^−1^; dA:8-oxo-dG (open squares): *v*_obs_ = 0.86 ± 0.03 nM min^−1^. (**C**) hpol κ (2 nM) extension from 14/18-mer DNA substrates (200 nM) in the presence of WRN exo (100 nM). The reaction was initiated upon addition of dGTP (100 μM) and MgCl_2_ (5 mM). (**D**) WRN-catalyzed exonucleolytic degradation of primer in the presence of competing pol activity was quantified and plotted as a function of time. Both pol and exo product bands were quantified and the amount of degraded primer calculated as a fraction of the total DNA in each lane. The data were analyzed by linear regression to estimate the rate of primer degradation for the linear portion of the velocity curve: dC:dG (closed circles): *v*_obs_ = 0.069 ± 0.009 nM min^−1^; dC:8-oxo-dG (open circles): *v*_obs_ = 0.29 ± 0.02 nM min^−1^; dA:dG (closed squares): *v*_obs_ = 2.7 ± 0.3 nM min^−1^; dA:8-oxo-dG (open squares): *v*_obs_ = 2.0 ± 0.1 nM min^−1^.

Next, we tested whether the isolated WRN exo domain could preferentially degrade 8-oxo-dG containing bp (Figure 4A). In the absence of hpol κ, the WRN^1-333^ exo domain exhibits a clear preference for the degradation of dA-containing mis-pairs regardless of whether 8-oxo-dG is present or unmodified dG. The rate of degradation was ∼0.9 nM min^−1^ for the two dA-containing mis-pairs, whereas the rate of degradation for dC:dG and dC:8-oxo-dG bp was 0.33 and 0.40 nM min^−1^, respectively (Figure 4B). The rate of WRN-catalyzed dA:8-oxo-dG degradation is ∼2-fold faster than dC:8-oxo-dG.

We also tested whether WRN^1-333^ exo activity could degrade primers when competing with hpol κ activity (Figure 4C). In the presence of hpol κ-catalyzed nucleotide insertion, the rate of exonucleolytic degradation of dA:dG bp by WRN^1–333^ is 2.7 nM min^−1^, while dA:8-oxo-dG bp are degraded at a rate of 2.0 nM min^−1^ (Figure 4D). Conversely, WRN^1-333^ exo activity degrades dC:dG and dC:8-oxo-dG bp at a rate of 0.07 and 0.30 nM min^−1^, respectively. Thus, WRN^1-333^ is 39-fold more active on dA:dG mis-pairs than dC:dG bp, and WRN^1–333^ degrades dA:8-oxo-dG bp 6.7-fold faster than dC:8-oxo-dG bp when hpol κ extension activity competes directly with the exonuclease. When considering WRN exo activity on 8-oxo-dG-containing bp within the context of polymerization by hpol κ, it is important to note that, on its own, hpol κ exhibits a 5-fold preference for extension from dA:8-oxo-dG mis-pairs compared to dC:8-oxo-dG bp (Table 5). This preferential extension may be due in part to the fact that dA:8-oxo-dG mis-pairs are geometrically similar to dA:dT bp since 8-oxo-dG is rotated about the glycosyl bond (*χ*) into a *syn* orientation to form a Hoogsteen-type bp with dA (57). Importantly, hpol κ extension from dC:8-oxo-dG bp is as efficient as extension from dA:8-oxo-dG when WRN is present in the reaction mixture (Table 5). Thus, WRN exo activity on dA:8-oxo-dG substrates could potentially impart additional accuracy by removing mis-pairs inserted by hpol κ during error-prone bypass of 8-oxo-dG and allowing extension from error-free dC:8-oxo-dG bp.

## DISCUSSION

TLS is an important mechanism for tolerating DNA damage and Y-family pols are central to the bypass of many DNA adducts (5–7). Y-family pol activity is regulated through a variety of mechanisms mostly involving interactions with the sliding clamp, but additional protein–protein interactions that modify Y-family pol TLS properties have been identified (39,44,58–68). Interactions with WRN have been shown to stimulate the *in vitro* activity of Y-family pols and this stimulation occurs on templates that contain DNA adducts (47,55). We were interested in studying the mechanism by which WRN altered the kinetics of polymerization by hpol κ in order to better understand potential ramifications upon TLS. We find that the rate constant for full-length extension by hpol κ on unmodified DNA is increased in the presence of WRN and that this stimulation can be mostly attributed to the RQC domain. We observe some stimulation of hpol κ extension by WRN constructs that contain the exo domain, indicative of a two-part (bipartite) functional interaction between the two proteins which depends on both the RQC and exo domains of WRN. At the level of single-nucleotide insertions, the RQC domain of WRN appears to be most important for enhancing hpol κ activity on undamaged DNA templates. The bipartite functional interaction between WRN and hpol κ involves a physical interaction based on pull-down assays. Also, co-localization of WRN and hpol κ in HeLa cells exposed to H_2_O_2_ is consistent with the idea that the physical/functional interaction observed with recombinant enzymes may be carried over into the cellular context.

As noted before, hpol κ is the most error-prone of the four human Y-family pols at bypass of the ubiquitous 8-oxo-dG lesion (20,38–39,41,56,69–71). The preferential insertion of dAMP opposite 8-oxo-dG by hpol κ can be attributed in part to interactions between Met-135 and the damaged template strand which stabilize 8-oxo-dG in the *syn* conformation, which promotes Hoogsteen-type bonding with an incoming dATP (20). Compared to other Y-family members, such as Dpo4 from *S. solfataricus* and pol η, hpol κ appears to lack side chains in the little finger domain which facilitate *anti*-oriented 8-oxo-dG through hydrogen bonding with the O^8^ oxygen of 8-oxo-dG (42). Mutating Leu-508 in the little finger domain of hpol κ to lysine partially restores the electrostatic contact with the lesion as it produces an enzyme that is much less error-prone at the insertion step opposite 8-oxo-dG, only preferring dATP ∼3.2-fold over dCTP (20). By way of contrast, Rev1 uses a protein-template-directed mechanism to act as a deoxycytidyl transferase during 8-oxo-dG bypass (71), and human pol iota (hpol ι) relies upon a narrowed active-site cleft to prevent base pairing between *syn*-oriented 8-oxo-dG and dATP (69), which results in dC:8-oxo-dG insertions being preferred 30-fold over dA:8-oxo-dG mis-pairs (70). Strangely enough, hpol ι inserts dCMP opposite 8-oxo-dG only ∼2-fold more effectively than dGMP (70), but it is still more accurate than hpol κ at bypass of 8-oxo-dG. This is perhaps due to the similarity noted between *syn-*oriented 8-oxo-dG and dT, as hpol ι is known to preferentially insert dGMP opposite template dT (72).

**Figure 5.**
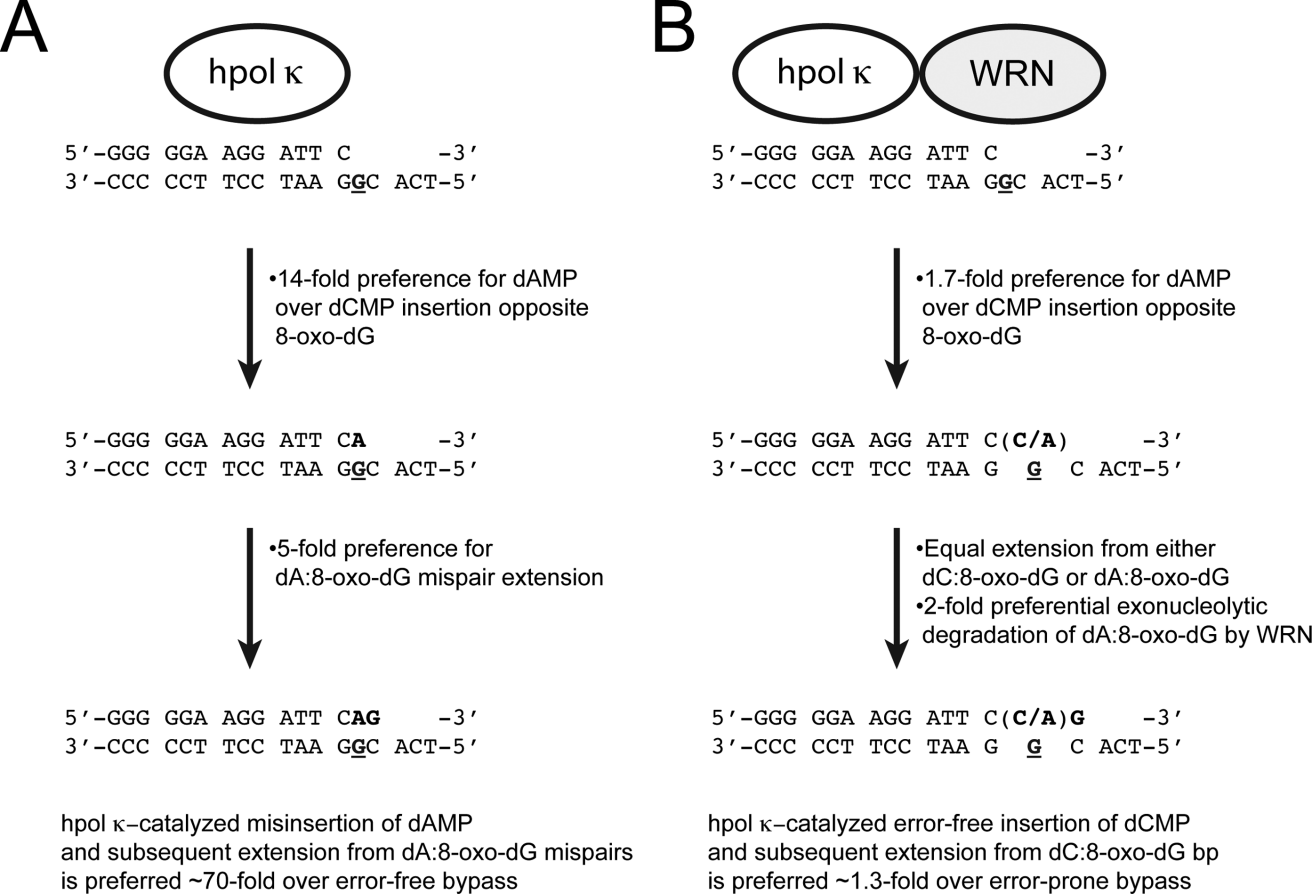
Model for WRN-dependent changes in hpol κ activity on 8-oxo-dG-modified template DNA. (**A**) The kinetic proficiencies of nucleotide selection by hpol κ at the insertion and extension steps are summarized [the position of 8-oxo-dG in the template is denoted with **G** in both panels (A) and (**B**)]. In isolation, hpol κ is ∼70-fold more efficient at error-prone insertion of dAMP opposite 8-oxo-dG and extension from dA:8-oxo-dG mis-pairs than it is error-free bypass of the lesion. (B) The kinetic proficiencies of nucleotide selection by hpol κ at the insertion and extension steps in the presence of WRN are summarized. The combined stimulation of accurate bypass by the WRN RQC domain and preferential degradation of dA:8-oxo-dG mis-pairs by WRN exo result in a slight (1.3-fold) preference for error-free bypass of 8-oxo-dG.

Our kinetic analyses indicate that WRN attenuates the error-prone nature of hpol κ-catalyzed bypass of 8-oxo-dG through a functional interaction involving the RQC and exo domains of WRN. WRN constructs possessing the RQC domain stimulate accurate insertion of dCMP opposite 8-oxo-dG, primarily by increasing *k*_cat_ and *k*_pol_ (Tables 2 and 4). The exo domain of WRN preferentially degrades dA:8-oxo-dG mis-pairs but allows unperturbed extension of dC:8-oxo-dG bp (Figure 4). To calculate the total effect of WRN on error-free bypass by hpol κ we multiplied 5.2 min^−1^ μM^−1^ (the specificity constant for dCMP insertion opposite 8-oxo-dG in the presence of full-length WRN) by 0.14 min^−1^ μM^−1^ (the specificity constant for extension from dC:8-oxo-dG bp in the presence of WRN), which yields a value of 0.73 min^−1^ μM^−1^. This value estimates the efficiency of accurate bypass (i.e. both insertion and extension steps) by hpol κ in the presence of WRN. For error-prone bypass we multiplied 8.9 min^−1^ μM^−1^ (the specificity constant for dAMP insertion opposite 8-oxo-dG in the presence of full-length WRN) by 0.13 min^−1^ μM^−1^ (the specificity constant for extension from dA:8-oxo-dG bp in the presence of WRN), which yields a value of 1.16 min^−1^ μM^−1^. This value estimates the efficiency of error-prone bypass (i.e. insertion and extension) by hpol κ in the presence of WRN. We then divided the value for dAMP insertion/extension (1.16 min^−1^ μM^−1^) by 2, since WRN exhibits a 2-fold preference for degrading dA:8-oxo-dG mis-pairs over dC:8-oxo-dG bp (Figure 4), which decreases the overall efficiency of error-prone bypass to 0.58 min^−1^ μM^−1^. Thus, the efficiency of error-free insertion/extension (0.73 min^−1^ μM^−1^) by hpol κ in the presence of WRN is actually 1.3-fold higher than the efficiency of error-prone insertion/extension/degradation (0.58 min^−1^ μM^−1^). WRN-mediated stimulation of hpol κ-catalyzed error-free bypass of 8-oxo-dG is even greater if we consider that degradation of dA:8-oxo-dG bp is 6.7-fold more rapid than dC:8-oxo-dG bp when hpol κ and WRN^1–333^ exo activities compete directly (Figure 4C and D). The cumulative effect of adding WRN to the reaction mixture is a 70-fold change in the kinetics of hpol κ-catalysis at the insertion and extension steps of bypassing 8-oxo-dG (Figure 5). While hpol κ remains relatively error-prone at bypass of 8-oxo-dG even in the presence of WRN, it is possible that additional interacting proteins, such as PCNA and RPA, could modulate the fidelity of lesion bypass further, as was observed for human pols λ and η (39).

The stimulation of hpol κ by WRN reported here is similar in many respects to previous studies with hpol η (47,55). In both cases, stimulation of TLS pol action appears to be driven through functional interactions primarily involving the WRN RQC. Pull-down experiments with recombinant enzymes indicate that both the WRN exo and RQC domains can physically interact with hpol κ (Figure 2A and B), again similar to results with hpol η (55). Moreover, we show that hpol κ and WRN co-localize in HeLa cells following exposure to oxidative damage (Figure 2C and D). A previous report from the Loeb group showed that WRN and hpol η co-localize following exposure to ultraviolet radiation sub-type C (UV-C) irradiation (47). This co-localization with hpol η was found to occur in the absence of either WRN helicase activity (i.e. with the WRN K577M mutant) or the WRN exo domain (47). Only WRN constructs possessing the RQC domain co-localized with hpol η in response to UV irradiation (47), consistent with our results showing that the RQC is the most important domain for stimulation of TLS pol activity.

The cellular role for hpol κ in the replication of 8-oxo-dG is not entirely clear, but it is interesting to consider how the functional interaction between WRN and hpol κ might influence 8-oxo-dG mutagenesis. A recent study used the *supF* mutagenesis assay to show that knock-down of hpol κ by siRNA reduced the frequency of G → T tranversions associated with mutagenic replication of 8-oxo-dG (43), suggesting that hpol κ copies unrepaired 8-oxo-dG in an error-prone fashion in human cells. Whether WRN modulates hpol κ bypass of 8-oxo-dG in cells remains to be seen, but WRN-deficient cells are known to accumulate mutations much faster than WRN-proficient cells (50,73). Moreover, WRN has been shown to modulate the efficiency of 8-oxo-dG bypass by hpol λ during MUTYH-mediated repair of dA:8-oxo-dG mis-pairs through a direct physical interaction between the core polymerase domain of hpol λ and either the helicase domain (a.a. 500-946) or C-terminal region (a.a. 949-1432) of WRN (51). However, neither the helicase nor the exo catalytic activity of WRN was required for enhancement of hpol λ activity. The stimulation of hpol λ by WRN was specific for 8-oxo-dG containing substrates and increased product formation ∼3-fold, similar to what we observe with hpols η and κ. One difference between our results and the study investigating WRN modulation of hpol λ is that WRN apparently stimulated both dCMP and dAMP insertion opposite 8-oxo-dG and did not appear to modify hpol λ fidelity one way or another. However, kinetic analysis of nucleotide selection by hpol λ was not performed and could yet reveal subtle changes in WRN-mediated stimulation of nucleotide insertion rates and/or affinities.

In summary, we have shown that WRN-mediated stimulation of hpol κ occurs through a physical and functional interaction that appears to involve the WRN exo and RQC domains. The modulation of hpol κ activity by WRN improves the fidelity of the TLS pol on both undamaged and 8-oxo-dG substrates by increasing the rate constant for polymerization (*k*_pol_). Co-localization of these two enzymes occurs in cells treated with H_2_O_2_. The cellular role for TLS pols in replication of 8-oxo-dG lesions leads us to conclude that WRN may act to suppress error-prone DNA synthesis by hpol κ following exposure to conditions that promote oxidative DNA damage. Experiments to examine the effects of WRN on hpol κ-mediated bypass of 8-oxo-dG in cells are ongoing.

## SUPPLEMENTARY DATA

Supplementary Data are available at NAR Online.

SUPPLEMENTARY DATA

## References

[1] Geacintov N., Broyde S. (2010). The Chemical Biology of DNA Damage.

[2] Miller J.A. (1998). The metabolism of xenobiotics to reactive electrophiles in chemical carcinogenesis and mutagenesis: a collaboration with Elizabeth Cavert Miller and our associates. Drug Metab. Rev..

[3] Friedberg E.C., Walker G.C., Siede W., Wood R.D., Shultz R.A., Ellenberger T. (2006). DNA Repair and Mutagenesis.

[4] Kornberg A., Baker T.A. (1992). DNA Replication.

[5] Chang D.J., Cimprich K.A. (2009). DNA damage tolerance: when it's OK to make mistakes. Nat. Chem. Biol..

[6] Sale J.E., Lehmann A.R., Woodgate R. (2012). Y-family DNA polymerases and their role in tolerance of cellular DNA damage. Nat. Rev. Mol. Cell Biol..

[7] Yang W., Woodgate R. (2007). What a difference a decade makes: insights into translesion DNA synthesis. Proc. Natl Acad. Sci. U.S.A..

[8] Choi J.Y., Angel K.C., Guengerich F.P. (2006). Translesion synthesis across bulky N2-alkyl guanine DNA adducts by human DNA polymerase kappa. J. Biol. Chem..

[9] Ogi T., Mimura J., Hikida M., Fujimoto H., Fujii-Kuriyama Y., Ohmori H. (2001). Expression of human and mouse genes encoding polkappa: testis-specific developmental regulation and AhR-dependent inducible transcription. Genes Cells.

[10] Ogi T., Shinkai Y., Tanaka K., Ohmori H. (2002). Pol kappa protects mammalian cells against the lethal and mutagenic effects of benzo[*a*]pyrene. Proc. Natl Acad. Sci. U.S.A..

[11] Avkin S., Goldsmith M., Velasco-Miguel S., Geacintov N., Friedberg E.C., Livneh Z. (2004). Quantitative analysis of translesion DNA synthesis across a benzo[*a*]pyrene-guanine adduct in mammalian cells: the role of DNA polymerase kappa. J. Biol. Chem..

[12] Jia L., Geacintov N.E., Broyde S. (2008). The N-clasp of human DNA polymerase kappa promotes blockage or error-free bypass of adenine- or guanine-benzo[*a*]pyrenyl lesions. Nucleic Acids Res..

[13] Lin J.R., Zeman M.K., Chen J.Y., Yee M.C., Cimprich K.A. (2011). SHPRH and HLTF act in a damage-specific manner to coordinate different forms of postreplication repair and prevent mutagenesis. Mol. Cell.

[14] Lupari E., Ventura I., Marcon F., Aquilina G., Dogliotti E., Fortini P. (2012). Pol kappa partially rescues MMR-dependent cytotoxicity of *O*^6^-methylguanine. DNA Repair (Amst).

[15] Albertella M.R., Lau A., O'Connor M.J. (2005). The overexpression of specialized DNA polymerases in cancer. DNA Repair (Amst).

[16] Wang H., Wu W., Wang H.W., Wang S., Chen Y., Zhang X., Yang J., Zhao S., Ding H.F., Lu D. (2010). Analysis of specialized DNA polymerases expression in human gliomas: association with prognostic significance. Neuro Oncol..

[17] Wang Y., Seimiya M., Kawamura K., Yu L., Ogi T., Takenaga K., Shishikura T., Nakagawara A., Sakiyama S., Tagawa M. (2004). Elevated expression of DNA polymerase kappa in human lung cancer is associated with p53 inactivation: Negative regulation of POLK promoter activity by p53. Int. J. Oncol..

[18] Bavoux C., Leopoldino A.M., Bergoglio V., J O.W., Ogi T., Bieth A., Judde J.G., Pena S.D., Poupon M.F., Helleday T. (2005). Up-regulation of the error-prone DNA polymerase κ promotes pleiotropic genetic alterations and tumorigenesis. Cancer Res..

[19] Haracska L., Unk I., Johnson R.E., Phillips B.B., Hurwitz J., Prakash L., Prakash S. (2002). Stimulation of DNA synthesis activity of human DNA polymerase kappa by PCNA. Mol. Cell. Biol..

[20] Irimia A., Eoff R.L., Guengerich F.P., Egli M. (2009). Structural and functional elucidation of the mechanism promoting error-prone synthesis by human DNA polymerase kappa opposite the 7,8-dihydro-8-oxo-2′-deoxyguanosine adduct. J. Biol. Chem..

[21] Benz C.C., Yau C. (2008). Ageing, oxidative stress and cancer: paradigms in parallax. Nat. Rev. Cancer.

[22] Bohr V., Anson R.M., Mazur S., Dianov G. (1998). Oxidative DNA damage processing and changes with aging. Toxicol. Lett..

[23] Ames B.N. (1989). Endogenous oxidative DNA damage, aging, and cancer. Free Radic. Res. Commun..

[24] Marnett L.J. (2000). Oxyradicals and DNA damage. Carcinogenesis.

[25] Gedik C.M., Boyle S.P., Wood S.G., Vaughan N.J., Collins A.R. (2002). Oxidative stress in humans: validation of biomarkers of DNA damage. Carcinogenesis.

[26] Karihtala P., Soini Y. (2007). Reactive oxygen species and antioxidant mechanisms in human tissues and their relation to malignancies. APMIS.

[27] Malins D.C., Anderson K.M., Jaruga P., Ramsey C.R., Gilman N.K., Green V.M., Rostad S.W., Emerman J.T., Dizdaroglu M. (2006). Oxidative changes in the DNA of stroma and epithelium from the female breast: potential implications for breast cancer. Cell Cycle.

[28] Malins D.C., Polissar N.L., Gunselman S.J. (1996). Progression of human breast cancers to the metastatic state is linked to hydroxyl radical-induced DNA damage. Proc. Natl Acad. Sci. U.S.A..

[29] Lindahl T. (1993). Instability and decay of the primary structure of DNA. Nature.

[30] Kalam M.A., Haraguchi K., Chandani S., Loechler E.L., Moriya M., Greenberg M.M., Basu A.K. (2006). Genetic effects of oxidative DNA damages: comparative mutagenesis of the imidazole ring-opened formamidopyrimidines (Fapy lesions) and 8-oxo-purines in simian kidney cells. Nucleic Acids Res..

[31] Brieba L.G., Eichman B.F., Kokoska R.J., Doublié S., Kunkel T.A., Ellenberger T. (2004). Structural basis for the dual coding potential of 8-oxoguanosine by a high-fidelity DNA polymerase. EMBO J..

[32] Einolf H.J., Guengerich F.P. (2001). Fidelity of nucleotide insertion at 8-oxo-7,8-dihydroguanine by mammalian DNA polymerase delta. Steady-state and pre-steady-state kinetic analysis. J. Biol. Chem..

[33] Einolf H.J., Schnetz-Boutaud N., Guengerich F.P. (1998). Steady-state and pre-steady-state kinetic analysis of 8-oxo-7,8-dihydroguanosine triphosphate incorporation and extension by replicative and repair DNA polymerases. Biochemistry.

[34] Freisinger E., Grollman A.P., Miller H., Kisker C. (2004). Lesion (in)tolerance reveals insights into DNA replication fidelity. EMBO J..

[35] Furge L.L., Guengerich F.P. (1997). Analysis of nucleotide insertion and extension at 8-oxo-7,8-dihydroguanine by replicative T7 polymerase exo- and human immunodeficiency virus-1 reverse transcriptase using steady-state and pre-steady-state kinetics. Biochemistry.

[36] Hsu G.W., Ober M., Carell T., Beese L.S. (2004). Error-prone replication of oxidatively damaged DNA by a high-fidelity DNA polymerase. Nature.

[37] Krahn J.M., Beard W.A., Miller H., Grollman A.P., Wilson S.H. (2003). Structure of DNA polymerase beta with the mutagenic DNA lesion 8-oxodeoxyguanine reveals structural insights into its coding potential. Structure.

[38] Haracska L., Yu S.L., Johnson R.E., Prakash L., Prakash S. (2000). Efficient and accurate replication in the presence of 7,8-dihydro-8-oxoguanine by DNA polymerase eta. Nat. Genet..

[39] Maga G., Villani G., Crespan E., Wimmer U., Ferrari E., Bertocci B., Hubscher U. (2007). 8-oxo-guanine bypass by human DNA polymerases in the presence of auxiliary proteins. Nature.

[40] Rechkoblit O., Malinina L., Cheng Y., Kuryavyi V., Broyde S., Geacintov N.E., Patel D.J. (2006). Stepwise translocation of Dpo4 polymerase during error-free bypass of an oxoG lesion. PLoS Biol..

[41] Zang H., Irimia A., Choi J.Y., Angel K.C., Loukachevitch L.V., Egli M., Guengerich F.P. (2006). Efficient and high fidelity incorporation of dCTP opposite 7,8-dihydro-8-oxodeoxyguanosine by *Sulfolobus solfataricus* DNA polymerase Dpo4. J. Biol. Chem..

[42] Eoff R.L., Irimia A., Angel K.C., Egli M., Guengerich F.P. (2007). Hydrogen bonding of 7,8-dihydro-8-oxodeoxyguanosine with a charged residue in the little finger domain determines miscoding events in *Sulfolobus solfataricus* DNA polymerase Dpo4. J. Biol. Chem..

[43] Kamiya H., Kurokawa M. (2012). Mutagenic bypass of 8-oxo-7,8-dihydroguanine (8-hydroxyguanine) by DNA polymerase kappa in human cells. Chem. Res. Toxicol..

[44] Garg P., Burgers P.M. (2005). Ubiquitinated proliferating cell nuclear antigen activates translesion DNA polymerases eta and REV1. Proc. Natl Acad. Sci. U.S.A..

[45] Garg P., Stith C.M., Majka J., Burgers P.M. (2005). Proliferating cell nuclear antigen promotes translesion synthesis by DNA polymerase zeta. J. Biol. Chem..

[46] Haracska L., Johnson R.E., Unk I., Phillips B., Hurwitz J., Prakash L., Prakash S. (2001). Physical and functional interactions of human DNA polymerase eta with PCNA. Mol. Cell. Biol..

[47] Kamath-Loeb A.S., Lan L., Nakajima S., Yasui A., Loeb L.A. (2007). Werner syndrome protein interacts functionally with translesion DNA polymerases. Proc. Natl Acad. Sci. U.S.A..

[48] Bohr V.A., Cooper M., Orren D., Machwe A., Piotrowski J., Sommers J., Karmakar P., Brosh R. (2000). Werner syndrome protein: biochemical properties and functional interactions. Exp. Gerontol..

[49] Damerla R.R., Knickelbein K.E., Strutt S., Liu F.J., Wang H., Opresko P.L. (2012). Werner syndrome protein suppresses the formation of large deletions during the replication of human telomeric sequences. Cell Cycle.

[50] Das A., Boldogh I., Lee J.W., Harrigan J.A., Hegde M.L., Piotrowski J., de Souza Pinto N., Ramos W., Greenberg M.M., Hazra T.K. (2007). The human Werner syndrome protein stimulates repair of oxidative DNA base damage by the DNA glycosylase NEIL1. J. Biol. Chem..

[51] Kanagaraj R., Parasuraman P., Mihaljevic B., van Loon B., Burdova K., Konig C., Furrer A., Bohr V.A., Hubscher U., Janscak P. (2012). Involvement of Werner syndrome protein in MUTYH-mediated repair of oxidative DNA damage. Nucleic Acids Res..

[52] Machwe A., Lozada E., Wold M.S., Li G.M., Orren D.K. (2010). Molecular cooperation between the Werner syndrome protein and replication protein A in relation to replication fork blockage. J. Biol. Chem..

[53] Pagano G., Zatterale A., Degan P., d'Ischia M., Kelly F.J., Pallardo F.V., Kodama S. (2005). Multiple involvement of oxidative stress in Werner syndrome phenotype. Biogerontology.

[54] Phillips L.G., Sale J.E. (2011). The Werner's Syndrome protein collaborates with REV1 to promote replication fork progression on damaged DNA. DNA Repair (Amst).

[55] Maddukuri L., Ketkar A., Eddy S., Zafar M.K., Griffin W.C., Eoff R.L. (2012). Enhancement of human DNA polymerase η activity and fidelity is dependent upon a bipartite interaction with the Werner's syndrome protein. J. Biol. Chem..

[56] Haracska L., Prakash L., Prakash S. (2002). Role of human DNA polymerase kappa as an extender in translesion synthesis. Proc. Natl Acad. Sci. U.S.A..

[57] McAuley-Hecht K.E., Leonard G.A., Gibson N.J., Thomson J.B., Watson W.P., Hunter W.N., Brown T. (1994). Crystal structure of a DNA duplex containing 8-hydroxydeoxyguanine-adenine base pairs. Biochemistry.

[58] Bienko M., Green C.M., Crosetto N., Rudolf F., Zapart G., Coull B., Kannouche P., Wider G., Peter M., Lehmann A.R. (2005). Ubiquitin-binding domains in Y-family polymerases regulate translesion synthesis. Science.

[59] Bienko M., Green C.M., Sabbioneda S., Crosetto N., Matic I., Hibbert R.G., Begovic T., Niimi A., Mann M., Lehmann A.R. (2010). Regulation of translesion synthesis DNA polymerase eta by monoubiquitination. Mol. Cell.

[60] Guo C., Sonoda E., Tang T.S., Parker J.L., Bielen A.B., Takeda S., Ulrich H.D., Friedberg E.C. (2006). REV1 protein interacts with PCNA: significance of the REV1 BRCT domain *in vitro* and *in vivo*. Mol. Cell.

[61] Haracska L., Kondratick C.M., Unk I., Prakash S., Prakash L. (2001). Interaction with PCNA is essential for yeast DNA polymerase eta function. Mol. Cell.

[62] Kannouche P.L., Wing J., Lehmann A.R. (2004). Interaction of human DNA polymerase eta with monoubiquitinated PCNA: a possible mechanism for the polymerase switch in response to DNA damage. Mol. Cell.

[63] Ohashi E., Hanafusa T., Kamei K., Song I., Tomida J., Hashimoto H., Vaziri C., Ohmori H. (2009). Identification of a novel REV1-interacting motif necessary for DNA polymerase kappa function. Genes Cells.

[64] Sarkies P., Murat P., Phillips L.G., Patel K.J., Balasubramanian S., Sale J.E. (2011). FANCJ coordinates two pathways that maintain epigenetic stability at G-quadruplex DNA. Nucleic Acids Res..

[65] Szüts D., Marcus A.P., Himoto M., Iwai S., Sale J.E. (2008). REV1 restrains DNA polymerase zeta to ensure frame fidelity during translesion synthesis of UV photoproducts *in vivo*. Nucleic Acids Res..

[66] Wojtaszek J., Lee C.J., D'Souza S., Minesinger B., Kim H., D'Andrea A.D., Walker G.C., Zhou P. (2012). Structural basis of Rev1-mediated assembly of a quaternary vertebrate translesion polymerase complex consisting of Rev1, heterodimeric polymerase (Pol) zeta, and Pol kappa. J. Biol. Chem..

[67] Xing G., Kirouac K., Shin Y.J., Bell S.D., Ling H. (2009). Structural insight into recruitment of translesion DNA polymerase Dpo4 to sliding clamp PCNA. Mol. Microbiol..

[68] Zhuang Z., Johnson R.E., Haracska L., Prakash L., Prakash S., Benkovic S.J. (2008). Regulation of polymerase exchange between Poleta and Poldelta by monoubiquitination of PCNA and the movement of DNA polymerase holoenzyme. Proc. Natl Acad. Sci. U.S.A..

[69] Kirouac K.N., Ling H. (2011). Unique active site promotes error-free replication opposite an 8-oxo-guanine lesion by human DNA polymerase iota. Proc. Natl Acad. Sci. U.S.A..

[70] Vaisman A., Woodgate R. (2001). Unique misinsertion specificity of pol iota may decrease the mutagenic potential of deaminated cytosines. EMBO J..

[71] Zhang Y., Wu X., Rechkoblit O., Geacintov N.E., Taylor J.S., Wang Z. (2002). Response of human REV1 to different DNA damage: preferential dCMP insertion opposite the lesion. Nucleic Acids Res..

[72] Tissier A., McDonald J.P., Frank E.G., Woodgate R. (2000). poliota, a remarkably error-prone human DNA polymerase. Genes Dev..

[73] Von Kobbe C., May A., Grandori C., Bohr V.A. (2004). Werner syndrome cells escape hydrogen peroxide-induced cell proliferation arrest. FASEB J..

