# Supplementary material for: The Werner syndrome protein limits the error-prone 8-oxo-dG lesion bypass activity of human DNA polymerase kappa
Source: Nucleic Acids Res. 2014 Oct 7;42(19):12027–40. doi: 10.1093/nar/gku913 (PMC4231769; doi:10.1093/nar/gku913)
Supplement: SUPPLEMENTARY DATA [file supp_42_19_12027__index.html]

The Werner syndrome protein limits the error-prone 8-oxo-dG lesion bypass activity of human DNA polymerase kappa — SUPPLEMENTARY DATA 

# The Werner syndrome protein limits the error-prone 8-oxo-dG lesion bypass activity of human DNA polymerase kappa

## SUPPLEMENTARY DATA

**Files in this Data Supplement:**

- SUPPLEMENTARY DATA
