# Supplementary material for: The Werner syndrome protein limits the error-prone 8-oxo-dG lesion bypass activity of human DNA polymerase kappa
Source: Nucleic Acids Res. 2014 Oct 7;42(19):12027–40. doi: 10.1093/nar/gku913 (PMC4231769; doi:10.1093/nar/gku913)
Supplement: SUPPLEMENTARY DATA [file supp_gku913_nar-02366-d-2014-File007.docx]

**The Werner syndrome protein limits the error-prone 8-oxo-dG lesion bypass activity of human DNA polymerase kappa**

**Leena Maddukuri^†^, Amit Ketkar^†^, Sarah Eddy^†^, Maroof K. Zafar^†^, and Robert L. Eoff^†1^**

*From the ^†^Department of Biochemistry and Molecular Biology, University of Arkansas for Medical Sciences, Little Rock, AR, 72205-7199, U.S.A.*

Address correspondence to:

Robert L. Eoff

Department of Biochemistry & Molecular Biology

University of Arkansas for Medical Sciences

4301 W. Markham St.

Little Rock, Arkansas 72205-7199

Telephone: (501) 686-8343

Fax: (501) 686-8169

# *Running Title: Mechanism of WRN interactions with hpol κ*

E-mail: [RLEOFF@UAMS.EDU](mailto:martin.egli@vanderbilt.edu)

**Supporting Information**

**Contents**

**Figure S1.** SDS-PAGE analysis of WRN mutants used in pull-down assays.

**Figure S2.** DNA binding capacity of WRN^949-1092^ RQC construct.

**Figure S3.** Full-length extension by hpol κ in the presence of different WRN constructs.

**Figure S4.** Steady-state analysis of single-nucleotide extension of dA:dG mis-pairs by hpol κ in the presence of WRN^1-1432^.


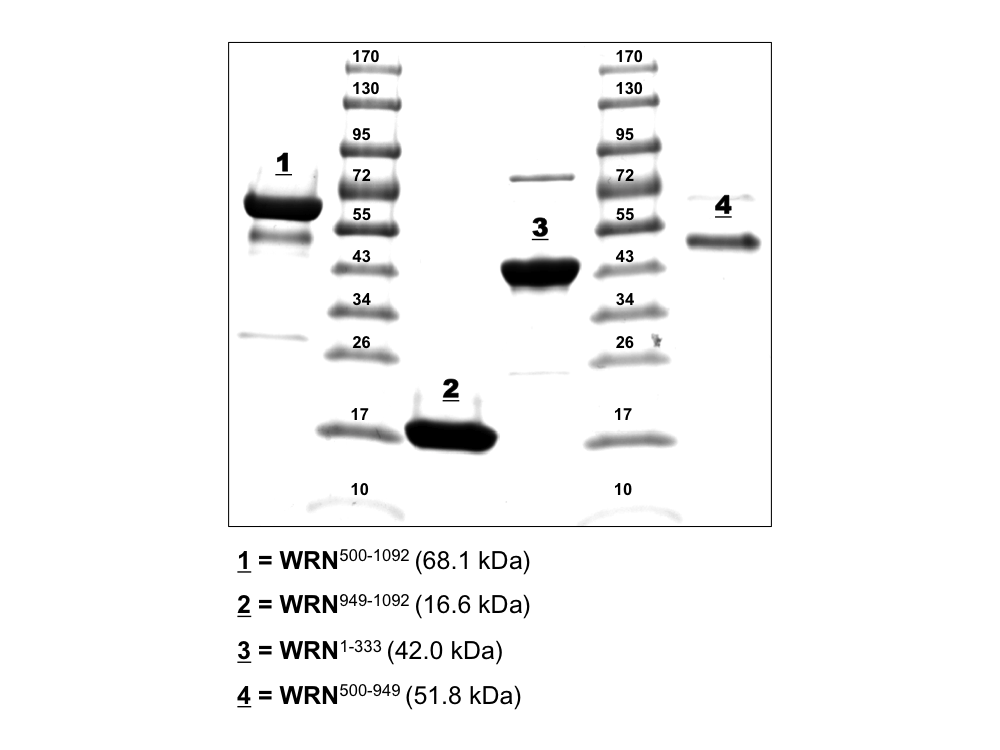


**Figure S1.** SDS-PAGE analysis of WRN mutants used in pull-down assays. The purity of the WRN constructs used in the GST-pull down experiments was assessed using SDS-PAGE followed by Coomassie blue staining.

**
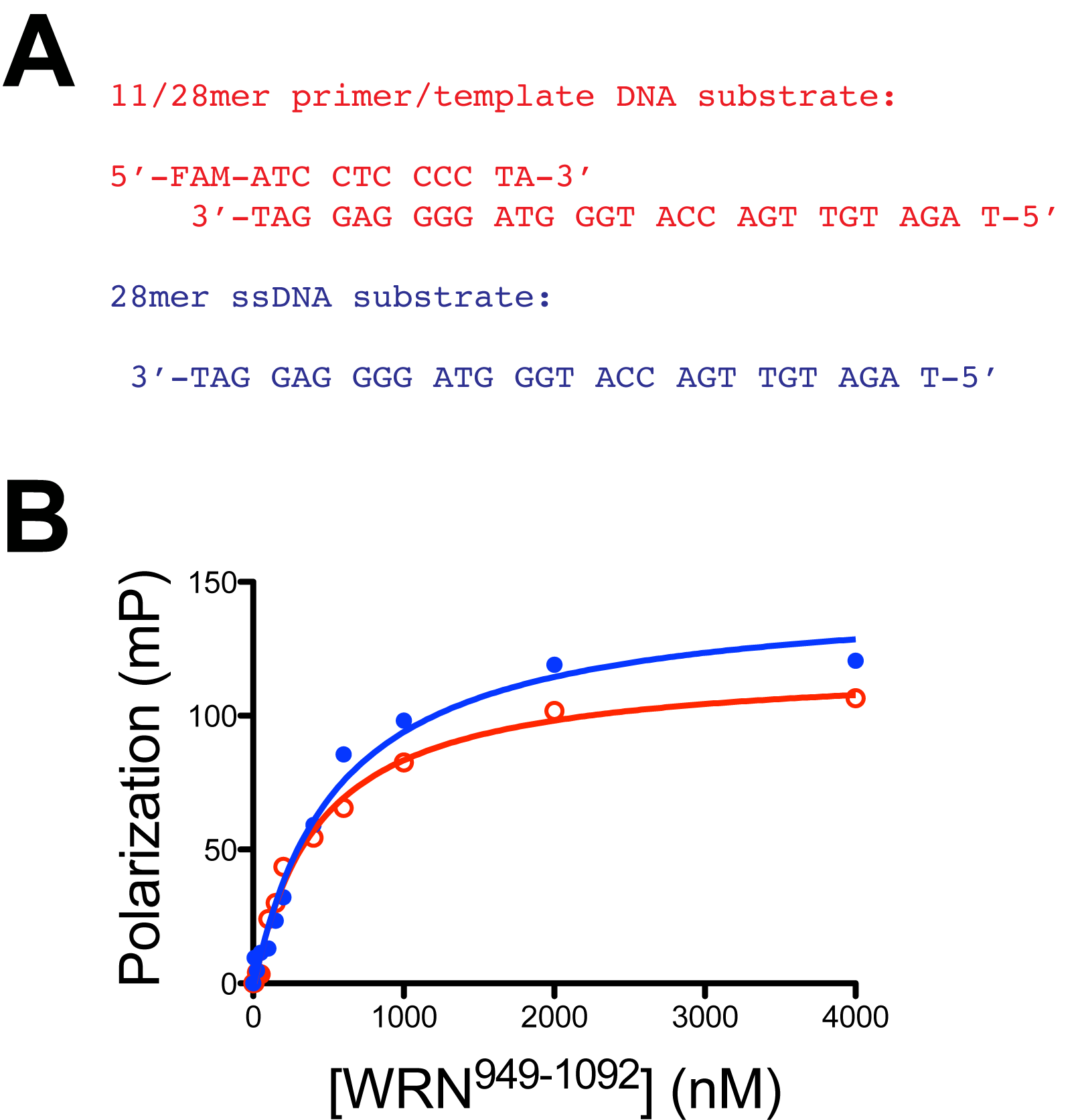
**

**Figure S2.** DNA binding capacity of WRN^949-1092^ RQC construct. **A.** Sequences of the substrates used to verify DNA binding by the WRN RQC construct (a.a. 949-1092). **B.** The change in fluorescence polarization was measured for increasing concentrations of WRN^949-1092^ titrated into a solution containing either 1 nM 28mer ssDNA (blue closed circles) or 11/28mer primer-template DNA (red open circles). The binding curves were fit to a quadratic equation to yield *K*_d,DNA_ values of 551 ± 109 nM and 418 ± 55 nM for 28mer and 11/28mer DNA substrates, respectively.

**
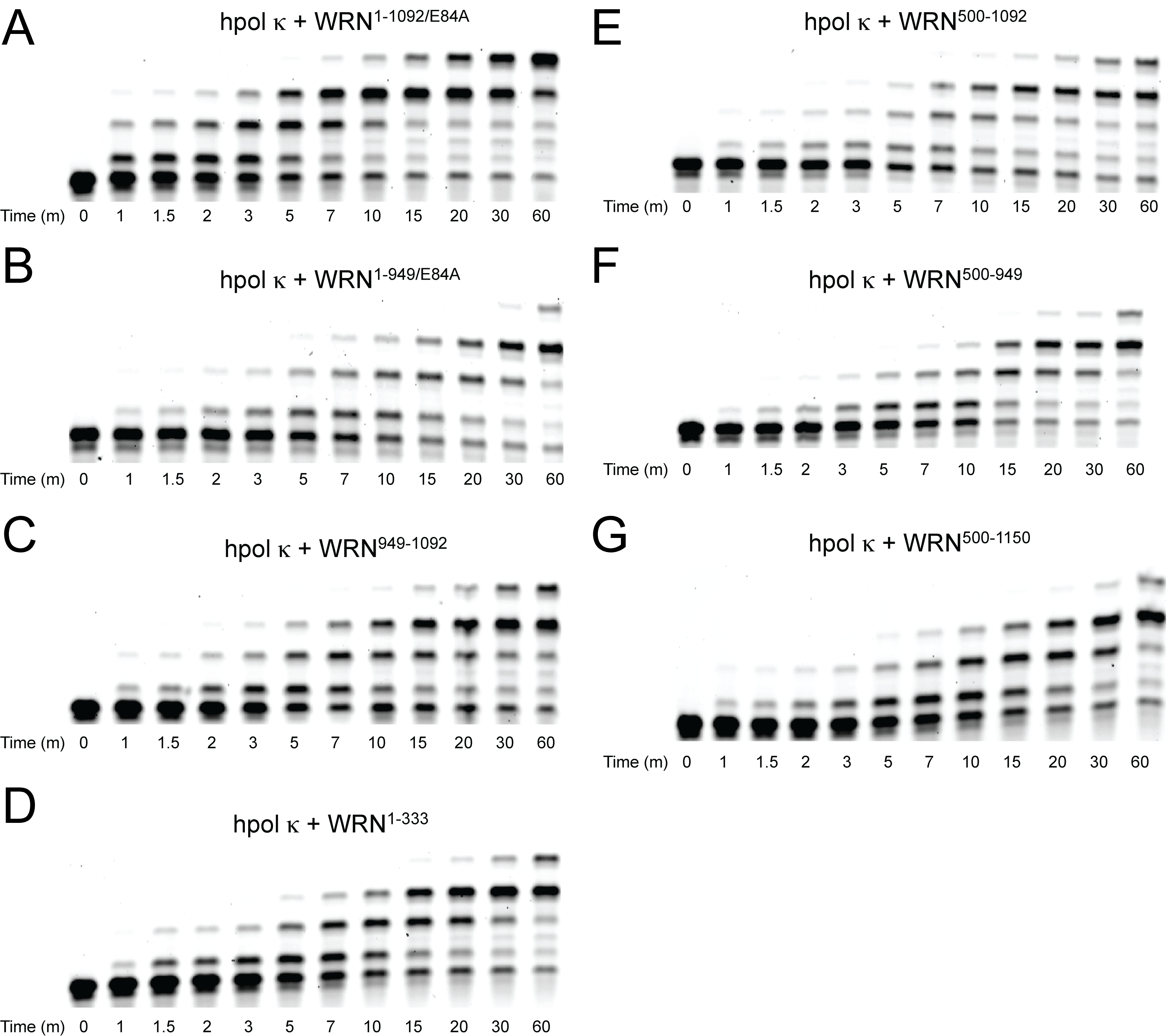
**

**Figure S3.** Full-length extension by hpol κ in the presence of different WRN constructs. DNA synthesis by hpol κ^19-526^ (2 nM) was monitored over time using a 13/18-mer primer-template DNA substrate (200 nM) in the absence of WRN and in the presence of 100 nM (**A**) WRN^1-1092/E84A^, (**B**) WRN^1-949/E84A^, (**C**) WRN^949-1092^, (**D**) WRN^1-333^, (**E**) WRN^500-1092^, (**F**) WRN^500-949^ and (**G**) WRN^500-1150^.

**
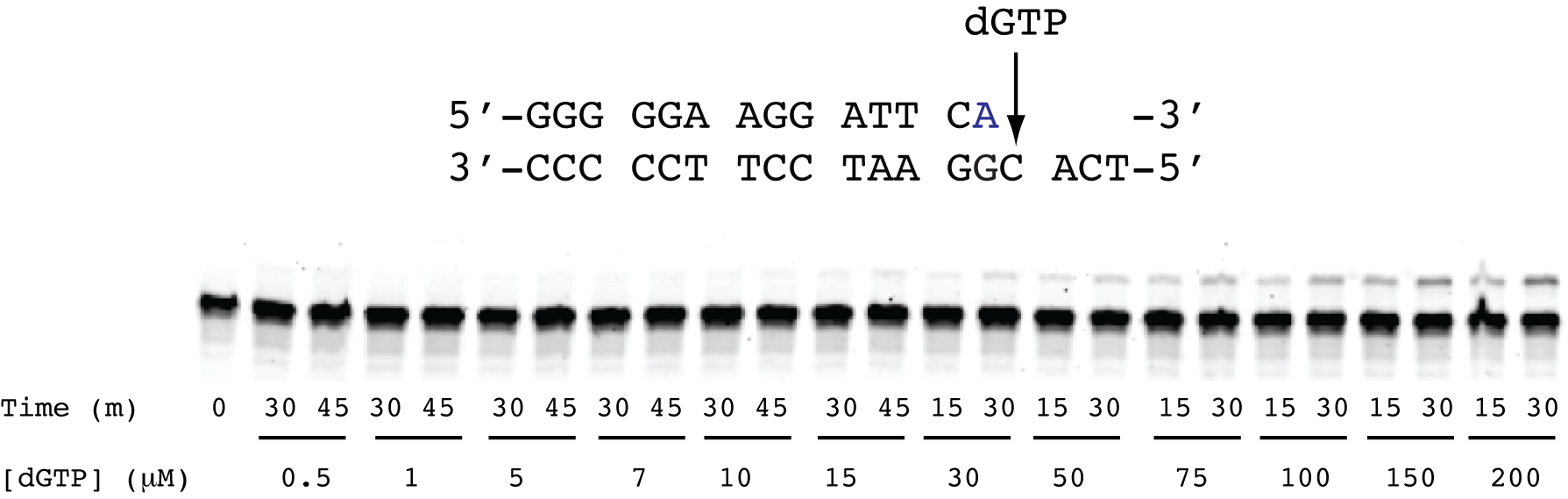
**

**Figure S4.** Steady-state analysis of single-nucleotide extension of dA:dG mis-pairs by hpol κ in the presence of WRN^1-1432^. Next-base extension by hpol κ^19-526^ (2 nM) was monitored using a 14A/18-mer primer-template DNA substrate (200 nM) in the presence of WRN^1-1432^ (100 nM) and varying concentrations of dGTP.
